# TNF-α/IL-1β-licensed hADSCs alleviate cholestatic liver injury and fibrosis in mice via COX-2/PGE2 pathway

**DOI:** 10.1186/s13287-023-03342-3

**Published:** 2023-04-24

**Authors:** Xiaoyu Luan, Peng Chen, Yaxin Li, Xinying Yuan, Longyu Miao, Pengyu Zhang, Qilong Cao, Xiaomin Song, Guohu Di

**Affiliations:** 1 School of Basic Medicine, Qingdao University, 308 Ningxia Road, Qingdao, 266071 China; 2 Institute of Stem Cell and Regenerative Medicine, School of Basic Medicine, Qingdao University, Qingdao, China; 3 Qingdao Haier Biotech Co. Ltd, Qingdao, China

**Keywords:** Adipose tissue-derived stem cells, TNF-α/IL-1β pretreatment, Bile duct ligation, Liver fibrosis, COX-2/PGE2 pathway

## Abstract

**Background:**

Adipose tissue-derived stem cell (ADSC) transplantation has been shown to be effective for the management of severe liver disorders. Preactivation of ADSCs enhanced their therapeutic efficacy. However, these effects have not yet been examined in relation to cholestatic liver injury.

**Methods:**

In the present study, a cholestatic liver injury model was established by bile duct ligation (BDL) in male C57BL/6 mice. Human ADSCs (hADSCs) with or without tumor necrosis factor-alpha (TNF-α) and interleukin-1beta (IL-1β) pretreatment were administrated into the mice via tail vein injections. The efficacy of hADSCs on BDL-induced liver injury was assessed by histological staining, real-time quantitative PCR (RT-qPCR), Western blot, and enzyme-linked immune sorbent assay (ELISA). In vitro, the effects of hADSC conditioned medium on the activation of hepatic stellate cells (HSCs) were investigated. Small interfering RNA (siRNA) was used to knock down cyclooxygenase-2 (COX-2) in hADSCs.

**Results:**

TNF-α/IL-1β preconditioning could downregulate immunogenic gene expression and enhance the engraftment efficiency of hADSCs. Compared to control hADSCs (C-hADSCs), TNF-α/IL-1β-pretreated hADSCs (P-hADSCs) significantly alleviated BDL-induced liver injury, as demonstrated by reduced hepatic cell death, attenuated infiltration of Ly6G + neutrophils, and decreased expression of pro-inflammatory cytokines TNF-α, IL-1β, C-X-C motif chemokine ligand 1 (CXCL1), and C-X-C motif chemokine ligand 2 (CXCL2). Moreover, P-hADSCs significantly delayed the development of BDL-induced liver fibrosis. In vitro, conditioned medium from P-hADSCs significantly inhibited HSC activation compared to that from C-hADSCs. Mechanistically, TNF-α/IL-1β upregulated COX-2 expression and increased prostaglandin E2 (PGE2) secretion. The blockage of COX-2 by siRNA transfection reversed the benefits of P-hADSCs for PGE2 production, HSC activation, and liver fibrosis progression.

**Conclusion:**

In conclusion, our results suggest that TNF-α/IL-1β pretreatment enhances the efficacy of hADSCs in mice with cholestatic liver injury, partially through the COX-2/PGE2 pathway.

**Supplementary Information:**

The online version contains supplementary material available at 10.1186/s13287-023-03342-3.

## Background

Cholestasis defines a frequent clinical condition mainly associated with the retention and accumulation of bile acids together with other toxic components in the hepatobiliary system [1, 2]. It is initiated by the extrahepatic obstruction of biliary tracts or intrahepatic impairment of bile excretion that eventually leads to parenchyma damage in the liver [3, 4]. Persistent cholestasis affects liver physiology and induces hepatocyte death, gradually evolving from severe inflammation [5] and substantial fibrosis [6] to malignant disease, triggering liver cirrhosis or carcinoma consequent to hepatic dysfunction [7, 8]. Although the mechanisms that drive the progression of such a disease have been widely studied [9–11], the treatments available for liver fibrosis in response to cholestatic injury are less developed. Overall, these highlight the need to explore new therapeutic approaches to reversing or delaying the aggravation of cholestatic liver disease to avoid remaining trapped in the dilemma of liver transplantation.

Adipose tissue-derived stem cells (ADSCs) with multi-differentiation potential have been considered promising candidates for cell therapy [12, 13]. By virtue of their immunomodulatory capacities, endogenous tissue repair potential, and other unique properties, ADSCs have been extensively utilized for the treatment of diverse diseases [14–17]. Previous studies have shown that ADSC transplantation could constitute an alternative therapeutic approach to combating or ameliorating hepatic disease owing to their positive effects on the modulation of inflammation and the alleviation of fibrosis formation [18–20]. Unfortunately, the survival crisis that mesenchymal stem cells (MSCs) inevitably suffer upon being transplanted into the host remains a major obstacle to translational success [21, 22]. The restrictions of allotransplantation and the inflammatory microenvironments of damaged sites give MSCs an inadequate survival rate in engraftment, further contributing to their insufficient efficacy [23].

Recently, various methods have been designed to modify transplanted MSCs to protect either their migration or proliferation abilities and enhance their curative effects [24–26]. It has been proven that pretreatment of MSCs with inflammatory cytokines at low concentrations orchestrates a feasible means of preadapting to many disease states, thus facilitating their immunoregulatory capacity [27–29]. Among the proinflammatory factors, tumor necrosis factor-alpha (TNF-α) is thought to exert a powerful synergistic effect in combination with interferon-gamma by mediating the anti-inflammatory functions and reducing the immunogenicity of MSCs [30, 31]. It is also worth mentioning that, in a murine model of intestinal ischemia and reperfusion, the administration of ADSCs treated with interleukin-1beta (IL-1β) decreased cellular apoptosis and promoted wound healing, which was reflected by improved paracrine function and the inhibited activation of inflammatory response pathways [32]. However, whether inflammatory cytokine pre-education could improve the therapeutic potential underlying the anti-inflammatory or anti-fibrotic effects of hADSCs on cholestasis-induced liver injury remains unclear.

In the present study, intravenous administration of pretreated hADSCs with the inflammatory cytokines TNF-α and IL-1β was used to investigate their efficacy and operative mechanism in the treatment of cholestatic liver disease. Our results demonstrated that pretreatment with TNF-α and IL-1β enhances the efficacy of hADSCs for BDL-induced liver injury predominantly by augmenting immunomodulatory activity via the cyclooxygenase-2/prostaglandin E2 (COX-2/PGE2) signaling pathway.

## Materials and methods

### Cell culture

hADSCs and LX-2 cells were cultured as in our previous descriptions [33, 34]. Briefly, hADSCs were cultured with hADSCs growth medium (HyCyte, Suzhou, China) in a humidified atmosphere with 5% CO_2_ at 37 °C. hADSCs from passages 4–6 were treated with or without TNF-α and IL-1β (10 ng/ml, PeproTech, Rocky Hill, NJ, USA) for 24, 48, and 72 h for the following experiment. Cultured hADSCs were characterized by flow cytometry (Cytoflex S, Beckman Coulter, Brea, CA, USA) using hADSC-specific markers. Adipogenic and osteogenic differentiation were constructed using hADSCs Adipogenic and Osteogenic Differentiation Kits (HyCyte) according to the manufacturer’s instructions. Immortalized LX-2 cells were maintained in Dulbecco’s modified Eagle’s medium (DMEM)-high glucose (HyClone, Cytiva, Marlborough, MA, USA) supplemented with 10% fetal bovine serum (FBS) (ExCell Bio, Shanghai, China) and 1% penicillin–streptomycin (Servicebio, Wuhan, China). Regarding the indirect co-culture system, conditioned media from hADSCs (C-hADSCs-CM) and P-hADSCs (P-hADSCs-CM) were collected as previously described [35], and the cultured medium of LX-2 cells was replaced with C-hADSCs-CM or P-hADSCs-CM in the presence of recombinant human transforming growth factor-beta1 (rhTGF-β) (2 ng/ml, PeproTech). The cells and culture supernatant were collected and stored at − 80 °C until the experiments were conducted.

### Transfection of hADSCs with small interfering RNA (siRNA)

hADSCs were transfected by COX-2 siRNA (sc-29279, Santa Cruz Biotechnology, CA, USA) combined with Lipofectamine 2000 (Invitrogen, Carlsbad, CA, USA), as specified in the manufacturer’s protocol. Fresh complete medium was replaced 6 h after transfection, and the cells were incubated for a further 48 h to make the silencing most effective.

### Animal model and treatments

Eight-week-old male C57BL/6 mice (20–22 g) were purchased from Beijing Vital River Laboratory Animal Technology Co., Ltd. (Beijing, China). All animal studies were performed in accordance with the regulations of the ARRIVE guidelines and approved by the Ethics Committee at the Medical College of Qingdao University (No.: QDU-AEC-2021165). A mouse model of cholestasis was induced by bile duct ligation (BDL) according to the method used in our previous study [34]. Briefly, after 5–6 h of fasting, the mice were anesthetized with 2% isoflurane (RWD Life Science, Shenzhen, China). By cutting the abdomen lengthwise, the common bile ducts were bluntly separated and doubly ligated with 3–0 silk sutures. Immediately after the operation, the mice (*n* = 5 per group) were randomly injected via the tail vein with hADSCs (C-hADSCs, 5 × 10^5^ cells/200 μl PBS), P-hADSCs (5 × 10^5^ cells/200 μl PBS), hADSCs transfected with si-NC (si-NC, 5 × 10^5^ cells/200 μl PBS), or hADSCs transfected with si-COX2 (si-COX2, 5 × 10^5^ cells/200 μl PBS). The PBS-transplanted group, as a vehicle control, was infused with equal doses of PBS. Mice in several groups were humanely euthanized with an overdose of pentobarbital sodium (150 mg/Kg, i.p.) 1, 2, and 7 days after transplantation. Blood serum was used for antibody detection by Enzyme-Linked Immune Sorbent Assay (ELISA). Liver tissues were collected and stored at − 80 °C for histology, immunohistochemistry, protein, and RNA analysis.

### Flow cytometric analysis (FACS)

After pretreatment with TNF-α and IL-1β or the absence thereof, the hADSCs were harvested by digestion with 0.05% trypsin/1 mM ethylenediaminetetraacetic acid (EDTA) (Specialty Media, Millipore, Billerica, MA, USA) and washed with PBS twice. The cell pellets were resuspended in PBS and incubated with anti-CD29-PE, anti-CD44-FITC, anti-CD34-FITC, anti-CD45-FITC, anti-CD80-PE, anti-CD86-PE, anti-HLA-ABC-FITC, and anti-HLA-DR-PE (all from BioLegend, San Diego, CA, USA) for about 30 min in the dark. The cytometry data were processed and analyzed using FlowJo software.

### In vivo bioluminescence imaging

hADSCs at passage 4 were transfected with an adenoviral vector containing a firefly luciferase reporter gene (Ad-Luc) (Shanghai Genechem Co., Ltd., Shanghai, China) to analyze the in vivo homing and survival rates. Transfected cells were injected via the tail vein at a concentration of 5 × 10^5^ cells per mouse directly after BDL surgery. Mice under anesthesia were injected intraperitoneally with D-luciferin and sodium salt (Yeasen, Shanghai, China) and imaged using an IVIS Lumina XRMS III in vivo imaging system (PerkinElmer, Waltham, MA, USA) at the indicated time points.

### ELISA and serum cytokine analysis

Serum was collected and cytokine levels were measured using an ELISA kit to determine the expression of alanine aminotransferase (ALT) (Servicebio), aspartate aminotransferase (AST) (Servicebio), PGE2 (Mlbio, Shanghai, China), TNF-α (Boster Biological Technology, Pleasanton, CA, USA), and IL-1β (Mlbio) according to the manufacturer’s instructions. After incubation, absorbance was finally read at 450 nm on a SpectraMax Absorbance Reader (Molecular Devices, Sunnyvale, CA, USA).

### Histopathological and immunohistochemical staining (IHC)

Liver samples from the left lateral lobes were fixed with 4% paraformaldehyde and embedded in paraffin. Sections stained with hematoxylin and eosin (H&E) and Sirius Red (Servicebio) were used to observe the pathological changes in liver tissues after deparaffinization and dehydration. Immunohistochemical staining was carried out by incubating the sections with cleaved-caspase 3 (C-caspase 3) (1:200, CST) and alpha-smooth muscle actin (α-SMA) (1:200, Abcam) antibody before visualization by color development with 3,3-diaminobenzidine (DAB) (Servicebio).

### Immunofluorescence staining (IF) and TUNEL assay

Mouse liver tissues were embedded in an optimal cutting temperature (OCT) compound (Servicebio) to make frozen sections. Serial sections of the tissues of 8 μm thickness were cut and used for the fluorescence observations. Double immunofluorescent staining was performed to evaluate immune cell infiltration with Ly6G antibody (Servicebio) and F4/80 antibody (Servicebio). The apoptosis of hepatocytes was detected using a terminal deoxynucleotidyl transferase dUTP nick-end labeling (TUNEL) assay from a commercial kit (Yeasen), following the manufacturer’s instructions. The nuclei were stained with 4',6-diamidino-2-phenylindole (DAPI) (Beyotime, Shanghai, China), and the positive cells were visualized using a fluorescence microscope.

### Western blot analysis

Protein samples were separated using sodium dodecyl sulfate (SDS)-polyacrylamide gel electrophoresis (PAGE) and electroblotted onto the nitrocellulose membranes (Millipore, Billerica, MA, USA). The membranes were then blocked with 5% fat-free milk powder dissolved in Tris-buffered saline/Tween 20 (TBST, 150 mM NaCl, 50 mM Tris–HCl, pH 7.5, 20% Tween 20) at room temperature for one hour and incubated with primary antibodies against COX-2 (1:1000, Abcam), α-SMA (1:1000, Abcam), β-actin (1:1000, ABclonal) or glyceraldehyde 3-phosphate dehydrogenase (GAPDH, 1:3000, KangChen Bio-tech) at 4 °C overnight. After three washes with TBST, the membranes were incubated with horseradish peroxidase (HRP)-conjugated goat anti-rabbit immunoglobulin G (IgG) antibody (1:4000, ABclonal) for one hour at room temperature. The membranes were washed again in TBST. Finally, the protein bands were imaged using an automatic chemiluminescence image analysis system (Tanon, Shanghai, China), and the expression levels of all target proteins were normalized to β-actin or GAPDH with ImageJ software (U.S. National Institutes of Health, Bethesda, MD, USA).

### Real-time quantitative PCR (RT-qPCR)

Total RNA was extracted with the FastPure Cell/Tissue Total RNA Isolation Kit (Vazyme, Nanjing, China), and complementary DNA was synthesized with a PrimeScript First Strand cDNA synthesis kit (TaKaRa, Dalian, China) according to the manufacturer’s protocol. Samples were run with Synergy Brands (SYBR) Premix Ex Taq (Vazyme) on Bio-Rad CFX96 Real-Time Systems (Bio-Rad, Hercules, CA), and the housekeeping gene GAPDH served as an internal control. The results were calculated using the 2-ΔΔCT method. All sequences of primers used for the PCR analysis are summarized in Table1.

**Table 1:** Gene-specific primers used in the RT-qPCR

Gene	Accession number	Primer sequences
m-GAPDH	NM_001289726.1	Forward: GCCACCCAGAAGACTGTGGAT
		Reverse: GGAAGGCCATGCCAGTGA
rh-GAPDH	NM_002046.7	Forward: CATGTTCGTCATGGGTGTGAA
		Reverse: GGCATGGACTGTGGTCATGAG
rh-HLA-DR	NM_019111.5	Forward: GGATGAGCCTCTTCTCAAGCA
		Reverse: CTTTTGCGCAATCCCTTGAT
rh-CD80	NM_005191.4	Forward: CTTCAACTGGAATACAACCAAGCA
		Reverse: TCATTCCTCCTTCTCTCTCTGCAT
rh-CD86	NM_175862.5	Forward: TGAACTGTCAGTGCTTGCTAACTTC
		Reverse: TGAATTCTTGGTTCTTAGCAAAACA
m-TNF-α	NM_013693.3	Forward: ACAAGGCTGCCCCGACTAC
		Reverse: TGGGCTCATACCAGGGTTTG
m-IL-1β	NM_008361.4	Forward: CTTTCCCGTGGACCTTCCA
		Reverse: CTCGGAGCCTGTAGTGCAGTT
m-IL-17	NM_010552.3	Forward: GACTCTCCACCGCAATGAAGAC
		Reverse: CTCTTCAGGACCAGGATCTCTTG
m-CXCL-1	NM_008176.3	Forward: CGCTTCTCTGTGCAGCGCTGCTGCT
		Reverse: AAGCCTCGCGACCATTCTTGAGTC
m-CXCL-2	NM_009140.2	Forward: CCTGGTTCAGAAAATCATCCA
		Reverse: CTTCCGTTGAGGGACAGC
m-TGF-β	NM_011577.2	Forward: CAACAATTCCTGGCGTTACCTT
		Reverse: CAAGAGCAGTGAGCGCTGAA
m-COL1A1	NM_007742.4	Forward: TGACTGGAAGAGCGGAGAGTACT
		Reverse: TTCGGGCTGATGTACCAGTTC
m-α-SMA	NM_007392.3	Forward: TGCCGAGCGTGAGATTGTC
		Reverse: CGTTCGTTTCCAATGGTGATC
rh-α-SMA	NM_001141945.2	Forward: GGTGACGAAGCACAGAGCAA
		Reverse: CAGGGTGGGATGCTCTTCAG
rh-COL1A1	NM_000088.4	Forward: CTGGATGCCATCAAAGTCTTCTG
		Reverse: CGCCATACTCGAACTGGAATC
rh-COX-2	NM_000963.4	Forward: AGCAGGCAGATGAAATACCAGTCT
		Reverse: ATACAGCTCCACAGCATCGATGT
rh-Annexin A1	NM_000700.3	Forward: TGACCGATCTGAGGACTTTGG
		Reverse: ACTCTGCGAAGTTGTGGATAGCT
rh-HGF	NM_000601.5	Forward: AGCATGTCAAGTGGAGTGAAAAAA
		Reverse: ACTCCAGGGCTGACATTTGATG
rh-TSG-6	NM_007115.4	Forward: TGCTACAACCCACACGCAAA
		Reverse: ACTCAGGTGAATACGCTGACCAT
rh-IDO	NM_002164.6	Forward: TGCAAGAACGGGACACTTTG
		Reverse: TGCCTTTCCAGCCAGACAA
rh-IL-1ra	NM_173842.3	Forward: ATTGAGCCTCATGCTCTGTTCTT
		Reverse: GAAGGCGAAGCGCTTGTC

### Statistical analysis

The results were presented as means ± standard deviation, and all statistical analyses were calculated using Prism 8.0 software (GraphPad, San Diego, CA, USA). Data were compared by the application of an unpaired Student’s *t* test and one-way analysis of variance (ANOVA). Differences with P values smaller than 0.05 were considered statistically significant.

## Results

### Effects of TNF-α/IL-1β pretreatment on the biological characteristics of hADSCs

**Fig. 1:**
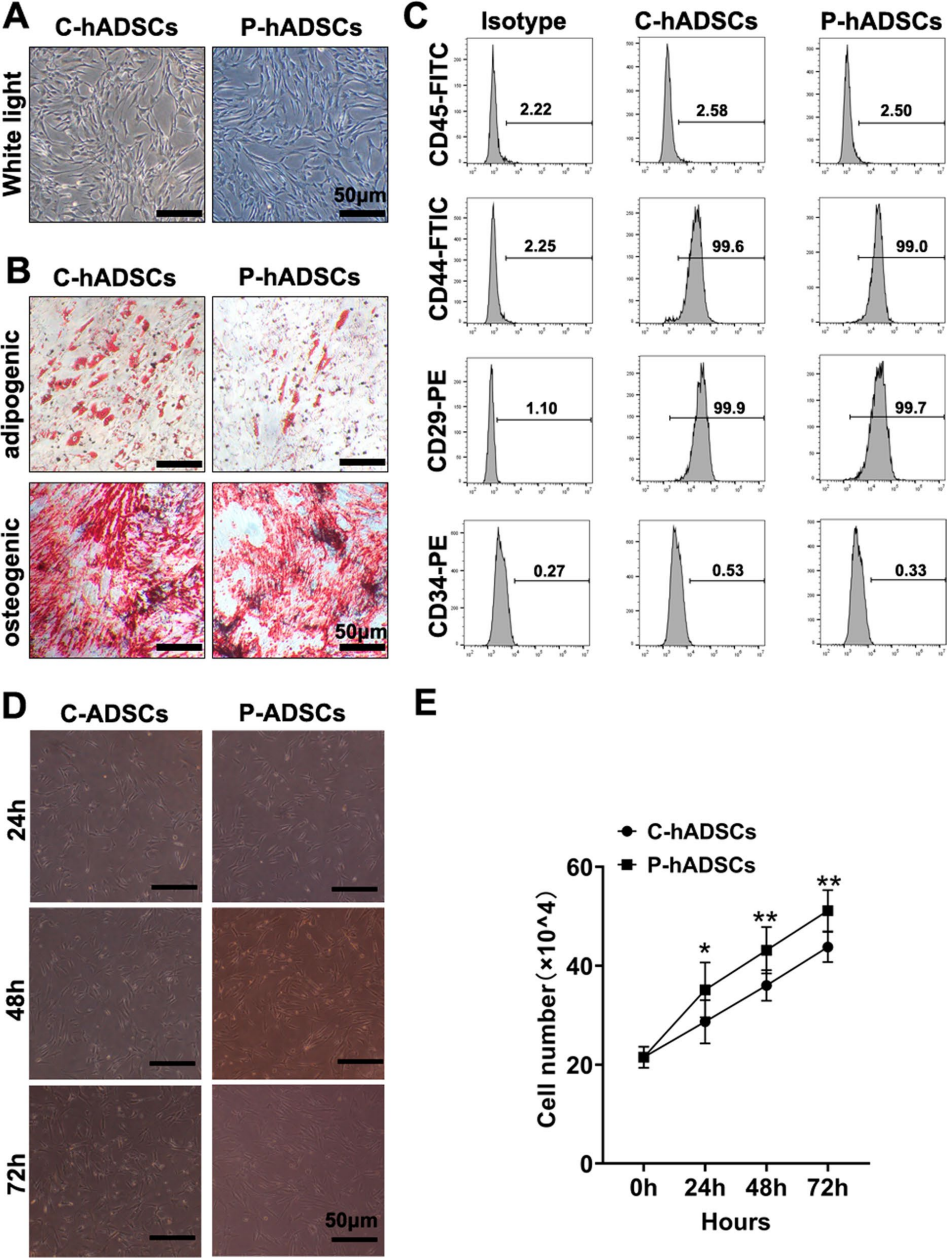
Characterization, phenotype identification, and proliferation of C-hADSCs and P-hADSCs. **a** Representative images of C-hADSCs (left) and P-hADSCs (right) morphology. **b** Representative images of Oil Red O staining and Alizarin Red staining of C-hADSCs and P-hADSCs after adipo-inductive and osteo-inductive incubation, respectively. **c** Expressions of surface markers (CD45, CD44, CD29, and CD34) were examined by flow cytometry of C-hADSCs and P-hADSCs. **d**,** e** Representative images and quantitative analysis of cell proliferation. Scale bars = 50 µm. Data were presented as the mean ± SD. *P < 0.05, **P < 0.01

hADSCs at passage 4 were characterized by a spindle-like and fusiform shape, exhibiting conspicuous refraction when being held in a vigorous state. After being pretreated with TNF-α and IL-1β for 24 h, the morphology of the hADSCs showed no significant differences between the two groups (Fig. 1a). In addition, Oil Red O staining and Alizarin Red staining were used to assess the pluripotency of the hADSCs. As shown in Fig. 1b, the staining results suggested that the osteogenic differentiation potential of the hADSCs was not affected, while the capacity for adipogenic differentiation was mildly impaired when primed by TNF-α/IL-1β. Furthermore, the flow cytometry analyses identified that TNF-α/IL-1β pretreatment had no influence on surface markers CD34, CD45, CD29, and CD44 of the hADSCs (Fig. 1c). Interestingly, the proliferative ability of the P-hADSCs was significantly enhanced compared to that of untreated hADSCs (Fig. 1d, e).

### TNF-α/IL-1β preconditioning enhanced the survival of hADSCs after transplantation

**Fig. 2:**
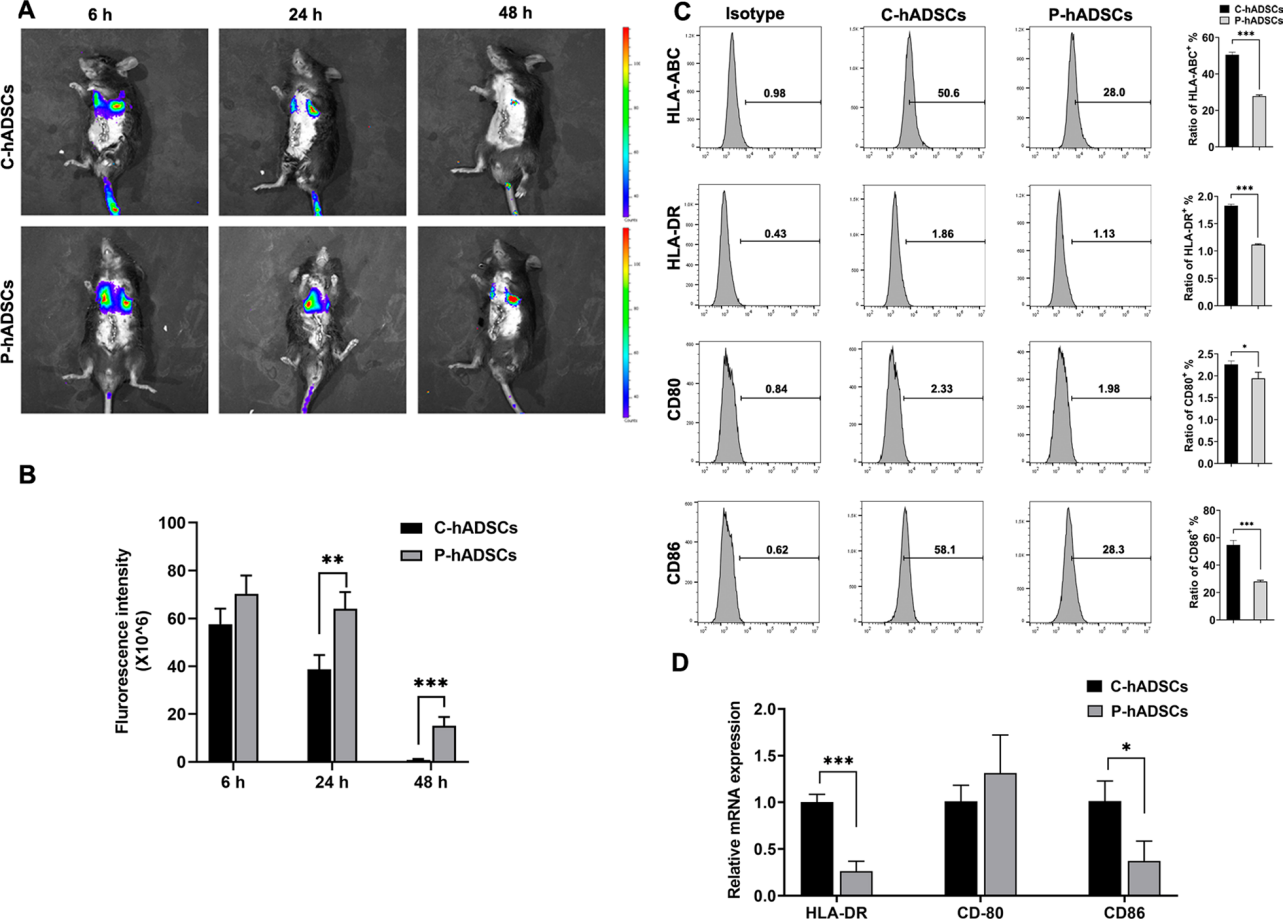
TNF-α/IL-1β preconditioning enhanced the survival of hADSCs after transplantation. **a** Biodistribution of C-hADSCs and P-hADSCs labeled by luciferase was injected intravenously after surgery. Representative IVIS images of mice injected with C-hADSCs (up) and P-hADSCs (down). **b** Quantification of the fluorescence intensity (*n* = 3 per group). **c** The positive ratio of HLA-ABC, HLA-DR, CD80, and CD86 was detected by flow cytometry (*n* = 3 per group). **d** RT-PCR analysis of HLA-DR, CD80, and CD86 (*n* = 3 per group). Scale bars = 50 µm. Data were presented as the mean ± SD. *P < 0.05, **P < 0.01, ***P < 0.001

To study the distribution patterns and survival conditions of transplanted cells in vivo, we performed bioluminescent IVIS (in vivo imaging system) imaging on C57BL/6 mice for observation. The preconditioned hADSCs or treatment-free controls were transfected with a lentivirus carrying the luciferase gene and injected into the mice via the tail vein. The intensity of the luciferase signal revealed that the systemically administrated cells could hone in on and aggregate in the lung but not the liver. Notably, at 24 h and 48 h post-administration, the durations of the luciferase signals in the mice treated with P-hADSCs showed significant increases compared with those of the control group (Fig. 2a, b), indicating that the survival times of P-hADSCs in the mice were significantly prolonged in comparison with those of C-hADSCs.

To further explore whether TNF-α/IL-1β pretreatment influences the immunogenicity of hADSCs, the expression of immunogenic markers (human leukocyte antigen (HLA)-ABC, HLA-DR) and costimulatory molecules (CD80, CD86) was analyzed. As shown in Fig. 2c, FACS analysis revealed a significant decrease in HLA-ABC and HLA-DR positive cells, with CD80 and CD86 following the same tendency (Fig. 2c). In addition, compared to the untreated group, P-hADSCs exhibited significantly lower HLA-DR and CD86 mRNA expression (Fig. 2d).

### TNF-α/IL-1β-primed hADSCs alleviated liver injury and inflammatory response in BDL Mice

**Fig. 3:**
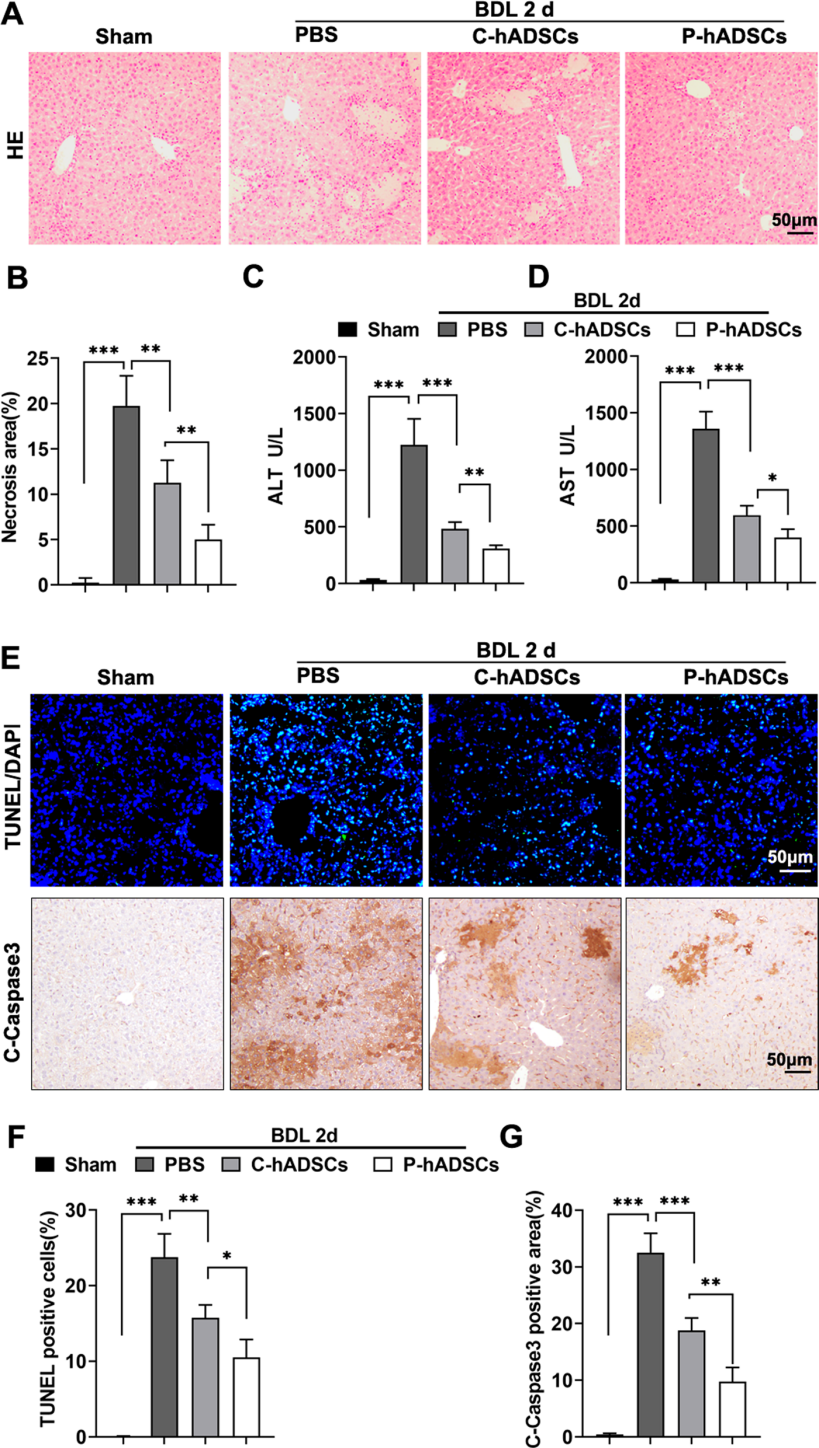
TNF-α/IL-1β-primed hADSCs alleviated liver injury in BDL mice. **a** Representative image of H&E staining of liver tissue in each group. **b** Quantification of hepatic necrosis area (*n* = 5 per group). **c**,** d** Detection of serum ALT and AST level (*n* = 5 per group). **e** Representative image of cell apoptosis was examined by TUNEL assays (upper) and immunostaining of C-caspase 3 (lower). **f** Quantification of TUNEL positive cells (*n* = 5 per group). **g** Quantification of C-caspase 3 positive area (*n* = 5 per group). Scale bars = 50 µm. Data were presented as the mean ± SD. *P < 0.05, **P < 0.01, ***P < 0.001

To investigate the efficacy of P-hADSCs on cholestatic liver injury, the mice were subjected to BDL followed by C-hADSCs (5 × 10^5^ cells per mouse) or P-hADSCs (5 × 10^5^ cells per mouse) administration through the tail vein immediately after operation. At 48 h after surgery, the mice in the PBS group showed increased hepatocyte damage compared to the sham group, as evidenced by an increased parenchymal necrosis area (Fig. 3a, b) and elevated serum AST and ALT levels. Infusion of hADSCs significantly reduced hepatic damage compared with the mice that received PBS. Mice in the P-hADSCs group showed significantly reduced hepatic necrosis, as well as lower AST and ALT levels, compared with mice in the C-hADSCs group (Fig. 3c, d). Moreover, BDL resulted in hepatocyte apoptosis and elevated C-caspase 3 activation. hADSC transplantation promoted liver function against BDL injury. P-hADSC administration led to a significant decrease in hepatic apoptosis compared with the mice given C-hADSCs 48 h after surgery (Fig. 3e–g).

**Fig. 4:**
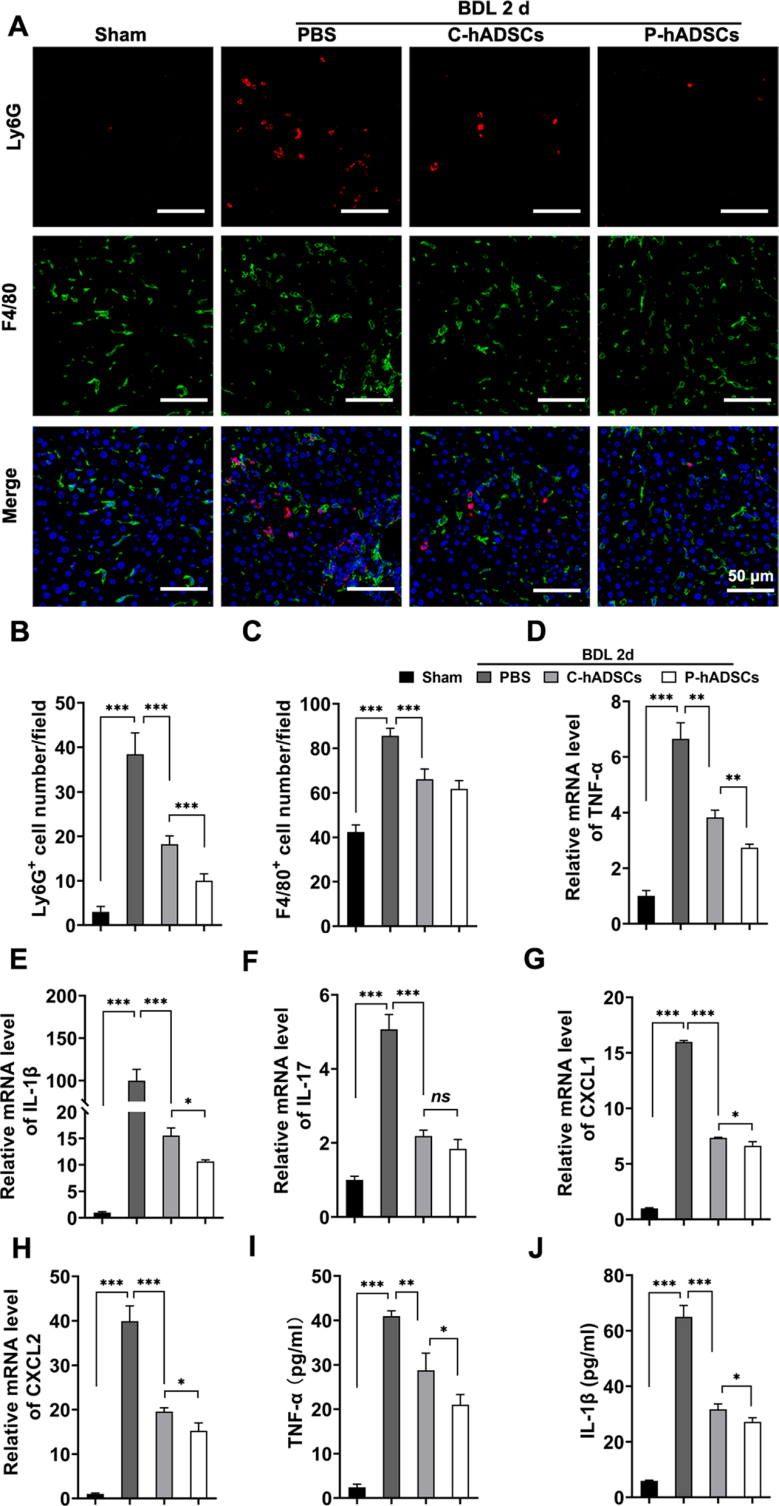
TNF-α/IL-1β-primed hADSCs alleviated inflammatory response in BDL mice.** a** Representative images of fluorescence staining of Ly6G and F4/80 in each group 48 h after surgery. **b**, **c** The quantitative analysis of Ly6G+ cells and F4/80+ cells in fluorescence staining (*n* = 5 per group). **d**–**h** The expression levels of mRNA for TNF-α, IL-1β, IL-17, CXCL1 and CXCL2 in liver tissue 48 h post-operation (*n* = 5 per group). **i**, **j** Serum levels of TNF-α and IL-1β were measured using ELISA (*n* = 5 per group). Scale bars = 50 µm. Data were presented as the mean ± SD. *P < 0.05, **P < 0.01, ***P < 0.001

Immune cell infiltration and inflammation response play an important role during the progression of liver fibrosis. Immunofluorescent staining revealed significantly less infiltration of Ly6G+ neutrophils, but not F4/80+ macrophages, in mice transplanted with P-hADSCs than in mice transplanted with C-hADSCs (Fig. 4a–c). Moreover, we further tested the proinflammatory cytokines TNF-α, IL-1β, and IL-17 and the chemokines CXCL1 and CXCL2 of the BDL mice that underwent different treatments. Infusion of hADSCs significantly reduced liver gene expression in TNF-α, IL-1β, CXCL1, and CXCL2, as well as serum TNF-α and IL-1β levels. The BDL mice that received P-hADSC infusion also had lower expression of TNF-α and IL-1β than the mice in the C-hADSC group (Fig. 4d–j).

### TNF-α/IL-1β-primed hADSCs alleviated BDL-induced liver fibrosis and HSC activation

**Fig. 5:**
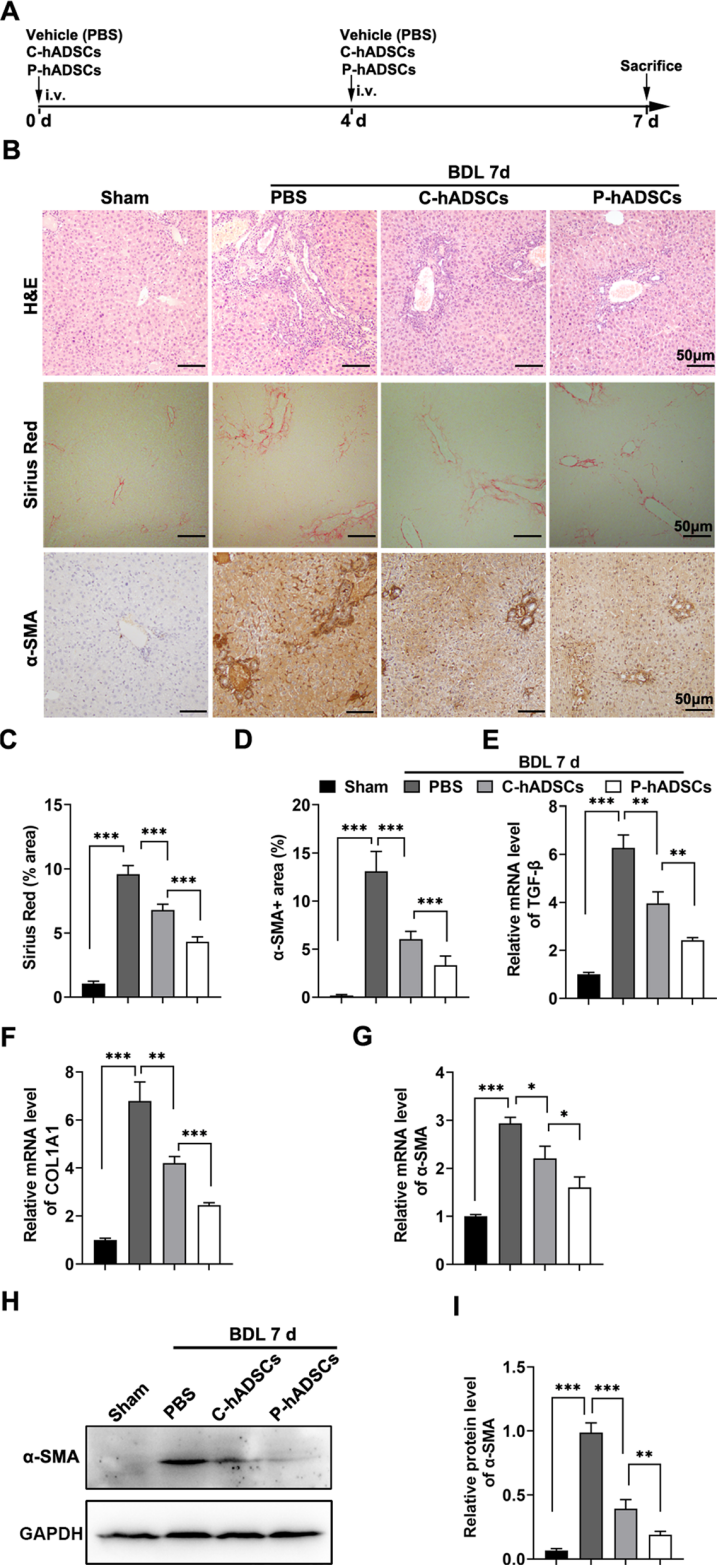
P-hADSCs alleviated the cholestatic liver fibrosis induced by bile duct ligation.** a** Schematic view of P-hADSCs therapy in a murine model of cholestatic liver fibrosis. **b** Representative images of H&E staining, Sirius red staining, and α-SMA immunohistochemical staining of liver sections from each group. **c** Quantification of Sirius red positive areas (*n* = 5 per group). **d** Quantification of α-SMA positive areas (*n* = 5 per group). **e**–**g** The expression levels of mRNA for TGF-β, COL1A1, and α-SMA in liver tissue 7 days post-operation. **h**, **i** Western blot and quantification of the protein expression for α-SMA in liver tissue. Scale bars = 50 µm. Data were presented as the mean ± SD. *P < 0.05, **P < 0.01, ***P < 0.001

To determine the effects of P-hADSCs on BDL-induced hepatic fibrogenesis, the BDL mice were administered P-hADSCs, C-hADSCs, or PBS by tail vein injection twice at 0 and 4 days, respectively, and killed 7 days after surgery (Fig. 5a). Histological examination of their liver sections was performed by H&E staining, Sirius Red staining, and α-SMA immunohistochemical staining. As expected, the BDL mice showed remarkable fibroplasia in the portal triad, increased collagen deposition, and an α-SMA positive area. The infusion of C-hADSCs significantly reduced collagen deposition and α-SMA expression compared with mice that received PBS. P-hADSC transplantation led to a significantly better improvement in collagen deposition and α-SMA expression compared to mice given hADSCs without TNF-α/IL-1β preconditioning (Fig. 5b–d). Meanwhile, RT-qPCR was performed to analyze the fibrogenic gene TGF-β, collagen type I alpha 1 chain (COL1A1), and α-SMA. Treatment with P-hADSCs remarkably downregulated the hepatic expression of the aforementioned profibrotic markers compared to mice treated with C-hADSCs (Fig. 5e–g). These results were further supported by α-SMA immunoblotting analysis (Fig. 5h, i; Additional file 1: Fig. S1).

**Fig. 6:**
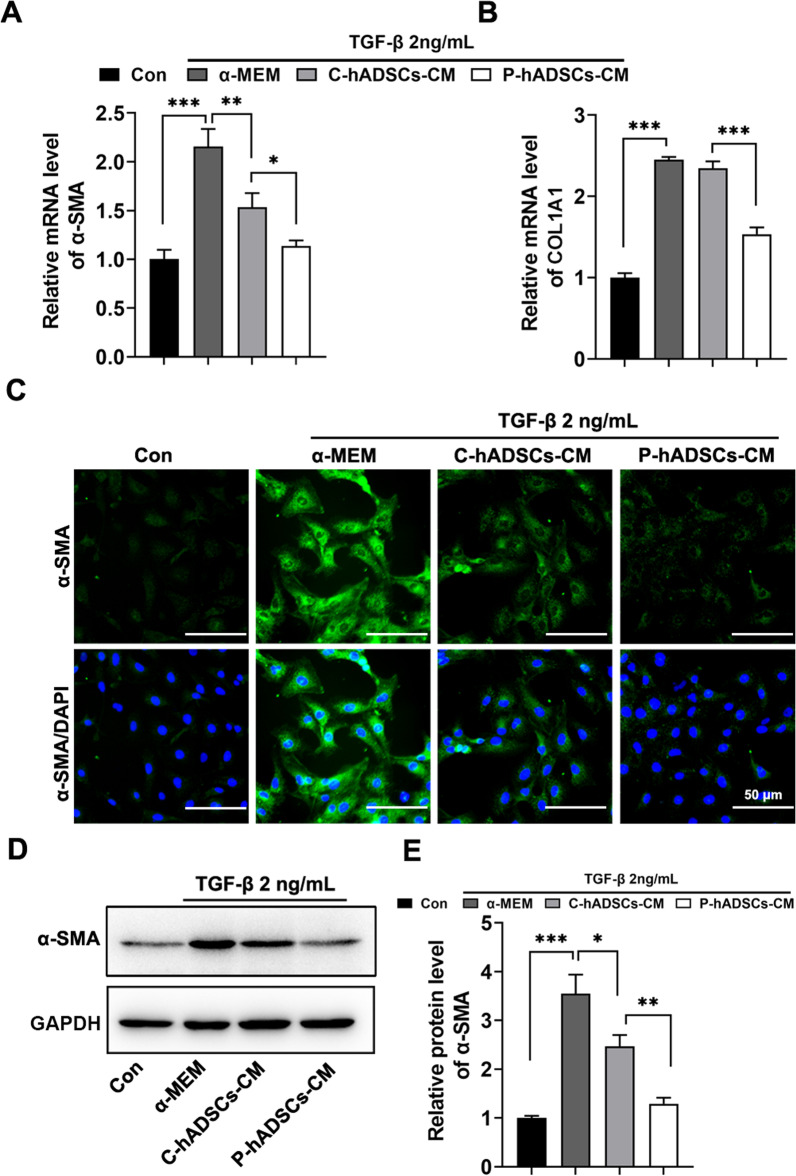
Conditioned medium from P-hADSCs (P-hADSCs-CM) inhibited HSC activation in vitro. LX-2 cells were cultured under conditioned medium from P-hADSCs (P-hADSCs-CM) or C-hADSCs (C-hADSCs-CM) in the presence of rhTGF-β for 48 h, with α-MEM used as control. **a**, **b** The expressions of mRNA for α-SMA and COL1A1 in LX-2 cells. **c** Representative immunofluorescence images of α-SMA staining from LX-2 cells. **d**, **e** Western blot analysis of α-SMA in LX-2 cells. Scale bars = 50 µm. Data were presented as the mean ± SD. *P < 0.05, **P < 0.01, ***P < 0.001

Furthermore, the effect of P-hADSCs on the activation of immortalized human HSCs (LX-2) was investigated. The LX-2 cells were treated with control medium (α-MEM), conditioned medium from C-hADSCs (C-hADSCs-CM), and conditioned medium from P-hADSCs (P-hADSCs-CM) together with rhTGF-β for 48 h. RT-qPCR analysis revealed that treatment of LX-2 cells with P-hADSCs-CM together with rhTGF-β resulted in decreased expression of the fibrotic genes α-SMA and COL1A1 compared to cells treated with C-hADSCs-CM (Fig. 6a, b). These results were further confirmed by α-SMA immunofluorescent staining and immunoblotting analysis, indicating that P-hADSCs-CM were able to inhibit LX-2 cell activation when the cells were cultured upon treatment with rhTGF-β (Fig. 6c–e; Additional file 1: Fig. S2).

### TNF-α/IL-1β pretreatment increased COX-2 expression and PGE2 secretion

**Fig. 7:**
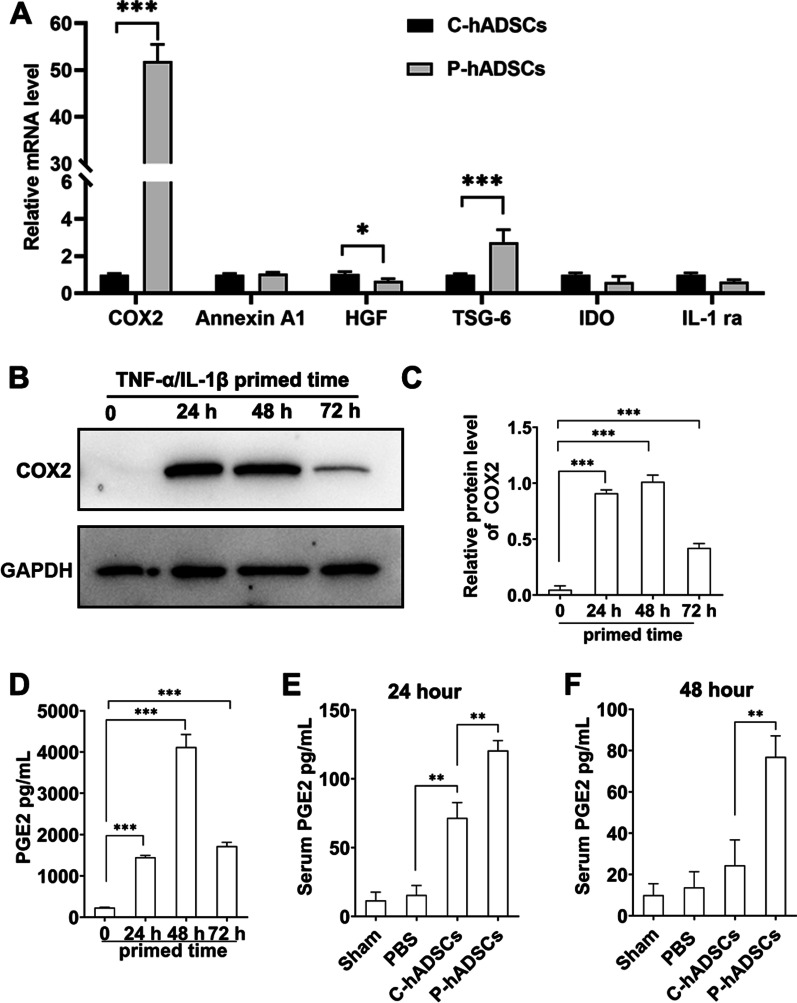
TNF-α/IL-1β pretreatment increased COX-2 expression and PGE2 secretion. **a** The expressions of mRNA for COX-2, Annexin A1, HGF, TSG-6, IDO and IL-1Ra in hADSCs after 24 h of TNF-α/IL-1β treatment. **b** The expression of COX-2 was determined by western blot analysis after hADSCs with 24 h, 48 h, and 72 h exposures of TNF-α/IL-1β. **c** Quantitative analysis of western blot. **d** The hADSCs supernatant PGE2 concentration were tested using ELISA (*n* = 3 per group). **e**, **f** PGE2 level in serum 24 and 48 h after cell transplantation were tested using ELISA (*n* = 5 per group). Data were presented as the mean ± SD. *P < 0.05, **P < 0.01, ***P < 0.001

Previous studies have reported that several immunomodulatory mediators, including COX-2, Annexin A1, hepatocyte growth factor (HGF), tumor necrosis factor-stimulated gene-6 (TSG-6), indoleamine 2,3-dioxygenase (IDO), and IL-1 receptor antagonist (IL-1Ra), contribute to the immunosuppressive function of MSCs [36]. To investigate the mechanism of TNF-α/IL-1β pretreatment in augmenting the immunomodulatory abilities of hADSCs, the above immune modulatory genes were analyzed. RT-qPCR results showed that expression levels of COX-2 and TSG-6, but not other genes, were upregulated when hADMSCs were treated with TNF-α/IL-1β for 24 h, with COX-2 expression increasing 50.95 ± 2.06-fold (Fig. 7a). In addition, the expression of COX-2 in hADSCs was measured after TNF-α/IL-1β pretreatment for different durations (0, 24, 48, and 72 h). Compared with the 0-h hADSCs, the 24-, 48-, and 72-h TNF-α/IL-1β-pretreated hADSCs showed significantly higher concentrations of COX-2 (Fig. 7b, c; Additional file 1: Fig. S3). Furthermore, the TNF-α/IL-1β pretreatment dramatically increased the PGE2 levels in the culture supernatant as well as the graft recipient serum compared with the hADSCs cultured without TNF-α /IL-1β (0-h hADSCs) (Fig. 7d–f).

### COX-2 silence reversed the efficacy of TNF-α/IL-1β-primed hADSCs on BDL-induced fibrosis

**Fig. 8:**
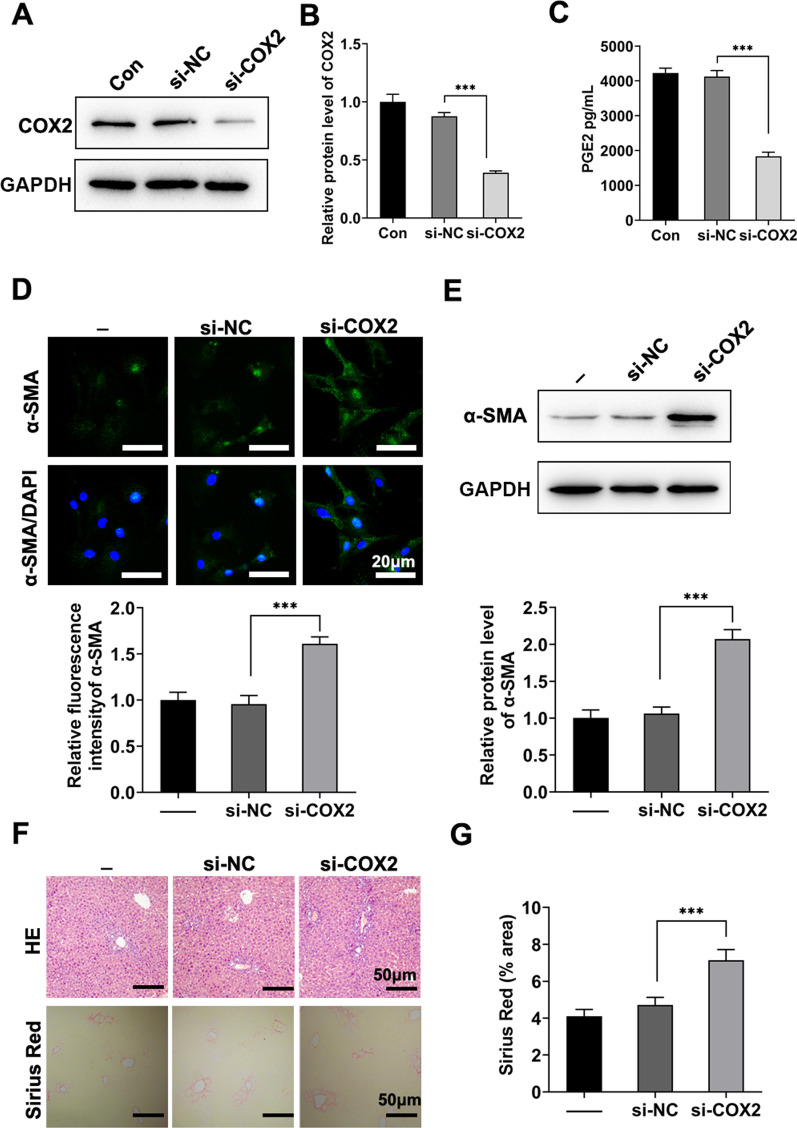
COX-2 silence reversed the efficacy of TNF-α/IL-1β-primed hADSCs on BDL-induced fibrosis. **a**, **b** hADSCs were transfected with si-NC or COX-2 siRNA and stimulated with TNF-α/IL-1β for 24 h. The knockdown of COX-2 was detected by western blotting. **c** The supernatant PGE2 concentration were tested using ELISA (*n* = 3 per group). **d** Representative immunofluorescence images and statistical analysis of α-SMA staining from LX-2 cells. **e** Western blot analysis of α-SMA in LX-2 cells. **f** Representative images of H&E staining and Sirius red staining. **g** Quantification of Sirius red positive areas (*n* = 5 per group). Scale bars = 50 µm. Data were presented as the mean ± SD. *P < 0.05, **P < 0.01, ***P < 0.001

To verify that TNF-α/IL-1β pretreatment improves the efficacy of hADSCs in cholestatic liver fibrosis through the COX-2/PGE2 pathway, COX-2 knockdown was performed using siRNA transfection. The knockdown efficiency of si-COX2 was verified via western blotting (Fig. 8a, b; Additional file 1: Fig. S4) and PGE2 secretion (Fig. 8c). Moreover, the inhibitory effect of P-ADSCs-CM on HSC activation was largely abolished by the knockdown of COX-2 (Fig. 8d, e; Additional file 1: Fig. S5). In addition, P-hADSCs with knocked-down COX-2 significantly reversed the anti-fibrotic effects in BDL mice, which was consistent with the intuitive results in vitro (Fig. 8f, g).

## Discussion

**Fig. 9:**
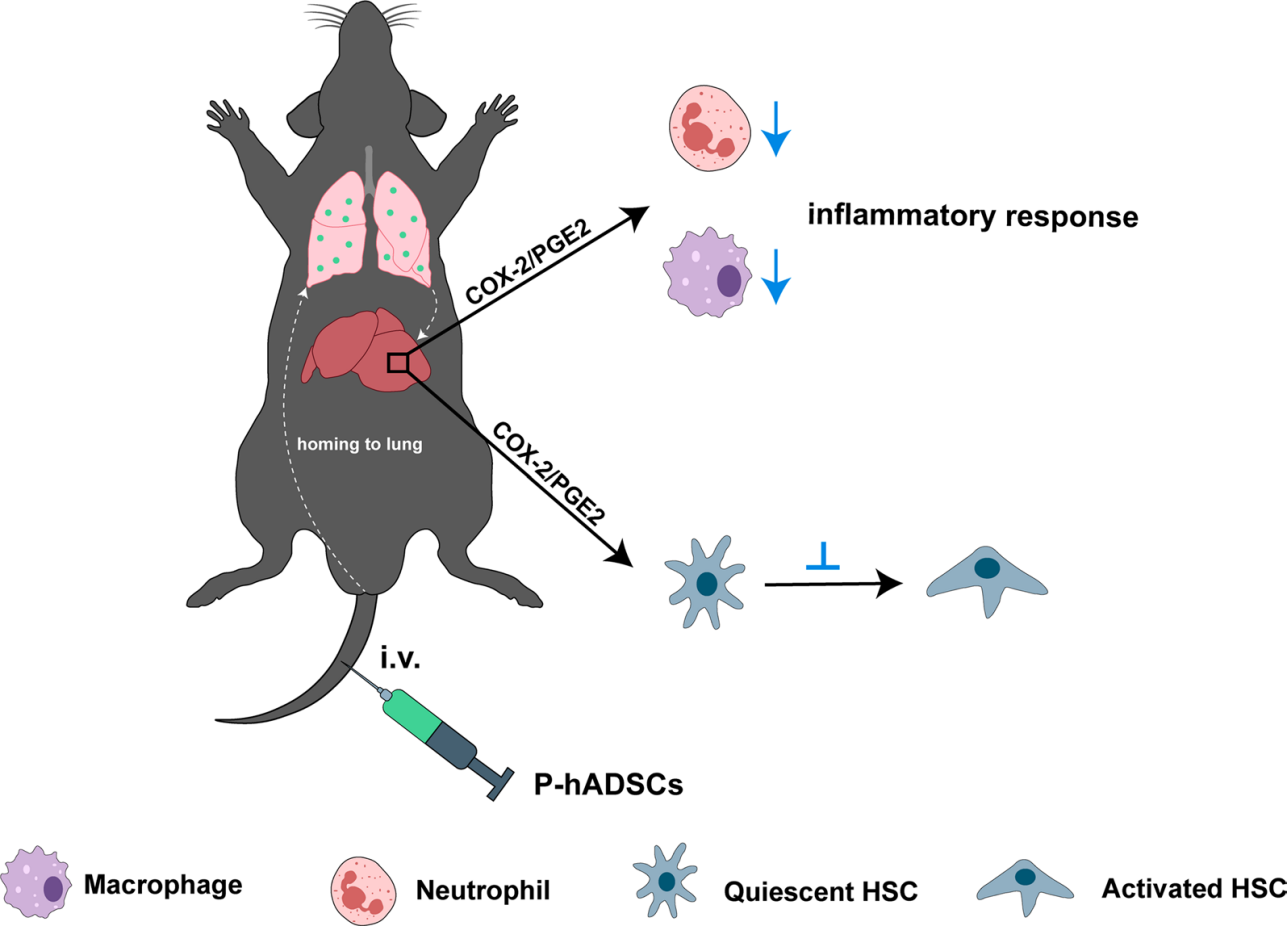
The schematic diagram showing the potential operative mechanisms of TNF-α/IL-1β-licensed hADSCs in BDL mice. This image was drawn by the authors

In this study, we provided novel insights regarding the enhanced efficacy and mechanism of TNF-α/IL-1β-pretreated hADSCs on cholestatic liver injury. First, TNF-α/IL-1β prelicense decreased the immunogenic-associated genes and improved the engraftment efficiency of hADSCs. Second, TNF-α/IL-1β pretreatment enhanced the efficacy of hADSCs in the improvement in hepatic function and liver fibrosis in BDL mice and increased the ability of hADSCs to inhibit HSC activation. Moreover, our data revealed that TNF-α/IL-1β upregulated COX-2 expression and PGE2 production, while COX-2 silence reversed the reinforced ability of TNF-α/IL-1β-pretreated hADSCs to attenuate liver fibrosis. These findings provide evidence that TNF-α/IL-1β pretreatment can enhance the efficacy of hADSCs in cholestatic liver injury via the COX-2/PGE2 pathway (Fig. 9).

Growing evidence suggests that the insufficient therapeutic efficiency of hADSCs could be related to the relatively limited survival rate of stem cells post-transplantation [37–40]. Anoikis has been reported as one of the main causes of this phenomenon [41]. Another suggestion is that widespread apoptosis of transplanted cells occurs in the host’s circulation or the recipient’s microenvironment during transplantation [23]. Despite their low immunogenicity, hADSCs can still be recognized and eliminated by the potent immune systems of recipient cells due to allogeneic transplantation [42–44]. The Fas ligand (FASL)-FAS pathway has been regarded as a highly correlated route of inducing immune rejection, thereby mediating the apoptosis of transplanted stem cells [45, 46]. In this study, the ratio of fluorescence signals in C-hADSCs decreased significantly 48 h after transplantation, indicating the loss of cells. Meanwhile, the TNF-α/IL-1β pretreatment group exhibited a significantly increased survival rate of transplanted hADSCs. Moreover, the signs of improvement could be associated with the reduction in surface costimulatory molecules. However, further research is needed to identify the underlying mechanisms.

Increased concentrations of bile acids are known to be an important stimulus of hepatocyte damage during cholestasis, causing chemoattractants to be released from necrotic hepatocytes [5, 47]. The consequent immune cascades initiated to promote the activation and recruitment of neutrophils ultimately led to an inflammatory response in the liver [48–50]. Studies have demonstrated that elevated levels of hepatocyte-specific proinflammatory cytokines, such as IL-1β [51], as well as enhanced expressions of CXCL1 [51, 52] and CXCL2 [5], are possibly mediated by the Farnesoid X Receptor (Fxr)/early growth response 1 (Egr1) pathway [53, 54]. Moreover, it has become clear that there is a complex mechanism of action among macrophages in regulating the fibrosis response. A milieu of continuous inflammation mediated the activation of HSCs, and macrophages were recruited to trigger their innate immune response. The broken balance between extracellular matrix (ECM) degradation and deposition promoted the initiation of liver fibrosis [55, 56]. It has been proven that hADSCs contribute to preventing or even eliminating further liver fibrogenesis by downregulating the expressions of inflammatory genes and inhibiting the activation of HSCs [18–20, 57, 58]. Consistently, our present data reveal that hADSC infusion ameliorated the pathological presentation of BDL-induced liver injury. In addition, we suggest that pretreatment with TNF-α and IL-1β directly enhances the immune regulation ability of hADSCs against hepatic inflammation and restrains the adverse consequence of cholestatic liver injury.

COX-2, an inflammation-induced isoform of cyclooxygenase, catalyzes the synthesis of prostaglandins and acts as an important mediator in MSC-mediated immune regulation [36, 59]. Among those prostaglandins, PGE2, a downstream lipid mediator generated by COX-2, has been described as a key suppressor of an immune response [60–62]. Recent studies have reported elevated COX-2/PGE2 expression when MSCs are exposed to inflammatory cytokines, such as IL-17 [27] and IL-1β [32]. Indeed, the COX-2/PGE2 axis has been demonstrated not only to have important access to mediating the immunosuppressive properties of MSCs [36, 63] but also to exert a decent anti-fibrotic effect on fibrosis diseases [64–66]. In the present study, our data revealed that TNF-α/IL-1β pretreatment dramatically stimulated COX-2 expression and PGE2 production in hADSCs. Notably, conditioned medium from TNF-α/IL-1β-pretreated hADSCs impeded the activation of HSCs, while COX-2 knockdown abolished the enhanced anti-fibrotic effects of hADSCs both in vivo and in vitro. These data support the hypothesis that COX-2/PGE2 signaling plays a critical role in mediating the enhanced therapeutic efficacy of TNF-α/IL-1β-pretreated hADSCs.

## Conclusion

In conclusion, this study suggests that TNF-α/IL-1β pretreatment could enhance the therapeutic effects of hADSCs on cholestatic liver injury. Moreover, TNF-α and IL-1β might promote hADSCs, partially by attenuating inflammatory response and inhibiting HSC activation by upregulating COX-2 expression and PGE2 production.

## Supplementary Information

**Additional file 1**. The full-length images of original gels.

## Data Availability

The data that support the findings during the current study are available from the corresponding author upon reasonable request.

## Abbreviations

ADSC: Adipose tissue-derived stem cell
BDL: Bile duct ligation
hADSC: Human adipose tissue-derived stem cell
TNF-α: Tumor necrosis factor-alpha
IL-1β: Interleukin-1beta
CXCL1: C-X-C motif chemokine ligand 1
CXCL2: C-X-C motif chemokine ligand 2
COX-2: Cyclooxygenase-2
PGE2: Prostaglandin E2
siRNA: Small interfering RNA
TGF-β: Transforming growth factor-beta
PBS: Phosphate-buffered saline
ALT: Alanine aminotransferase
AST: Aspartate aminotransferase
C-caspase 3: Cleaved-caspase 3
α-SMA: Alpha-smooth muscle actin
HLA-ABC: Human leukocyte antigen-ABC
HLA-DR: Human leukocyte antigen-DR
IL-17: Interleukin-17
HSC: Hepatic stellate cell
COL1A1: Collagen type I alpha 1 chain
HGF: Hepatocyte growth factor
TSG-6: Tumor necrosis factor-stimulated gene-6
IDO: Indoleamine 2,3-dioxygenase
IL-1Ra: IL-1 receptor antagonist
ECM: Extracellular matrix
