# Supplementary material for: TNF-α/IL-1β-licensed hADSCs alleviate cholestatic liver injury and fibrosis in mice via COX-2/PGE2 pathway
Source: Stem Cell Res Ther. 2023 Apr 24;14:100. doi: 10.1186/s13287-023-03342-3 (PMC10127380; doi:10.1186/s13287-023-03342-3)
Supplement: Supplementary file 1 — Additional file 1. The full-length images of original gels. [file 13287_2023_3342_MOESM1_ESM.docx]

**Supplementary figures and figure legends**

**
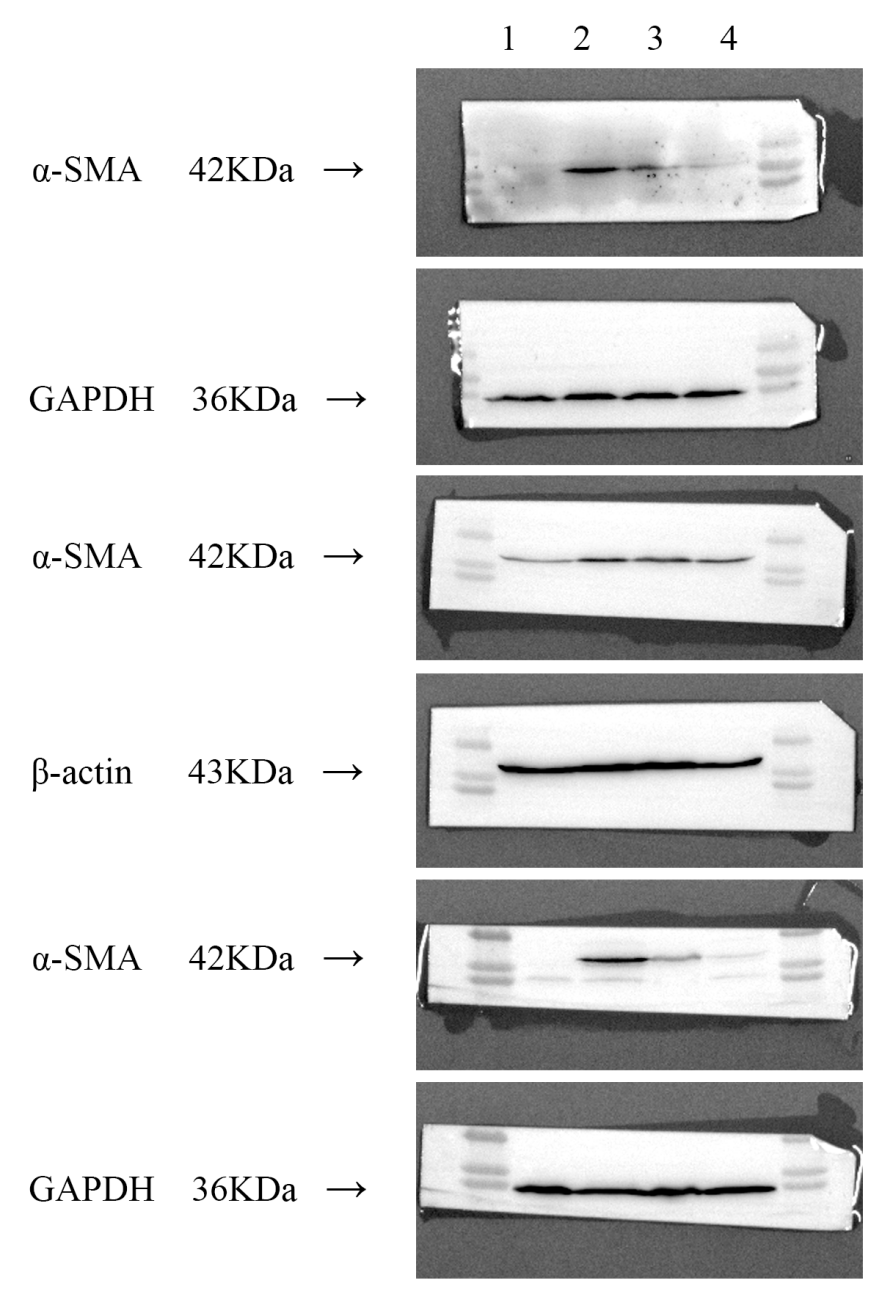
**

**Supplementary Figure 1.** **Full length gels of three independent western blots for Fig. 5H.** BDL mice treated with PBS, C-hADSCs or P-hADSCs, and liver tissues were collected for western blot. Lane 1: sham group (a midline laparotomy incision without bile duct ligation); Lane 2: PBS group (PBS injected by tail vein); Lane 3: C-hADSCs group (control hADSCs injected by tail vein); Lane 4: P-hADSCs group (TNF-α/IL-1β-pretreated hADSCs injected by tail vein).

**
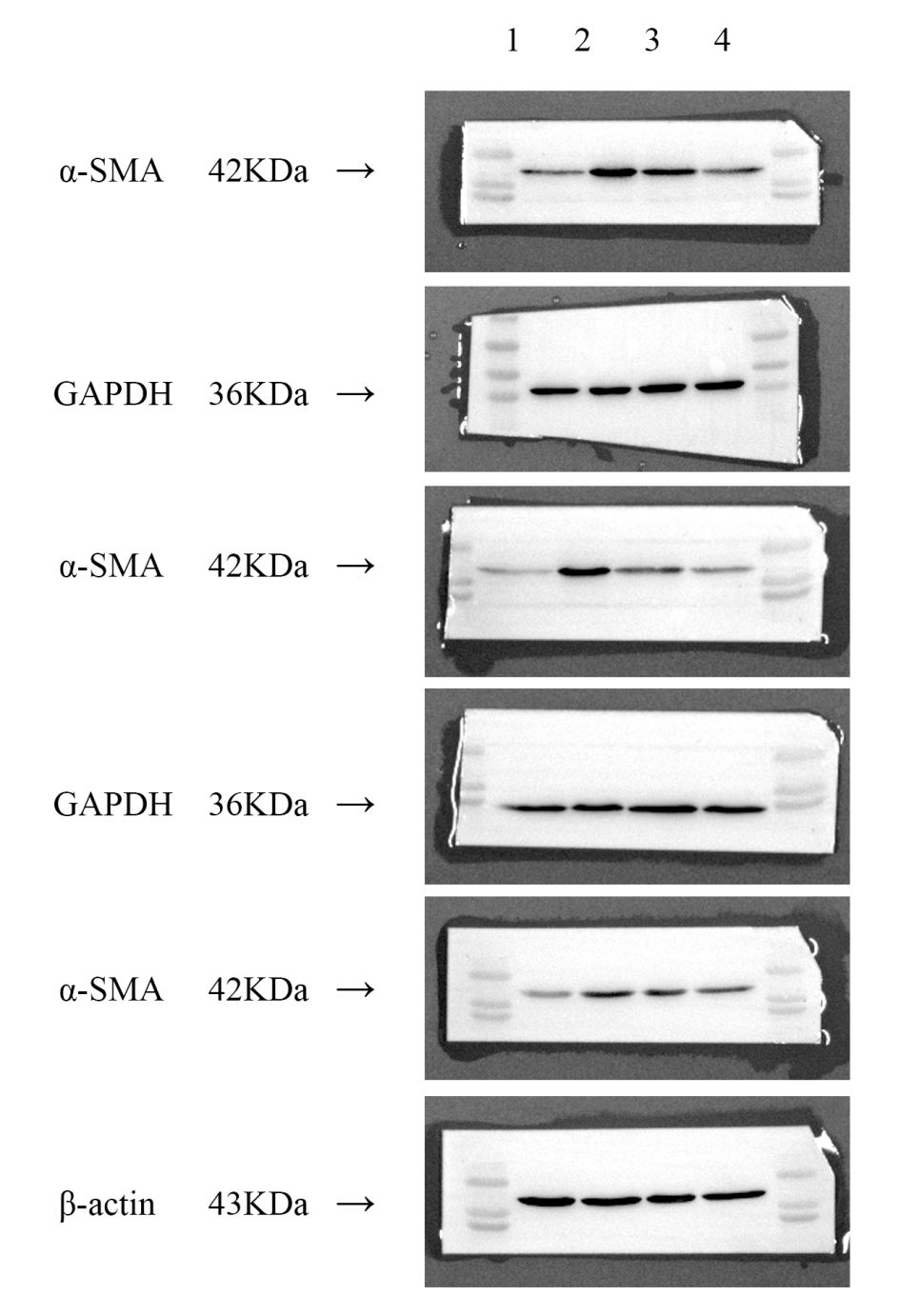
**

**Supplementary Figure 2. Full length gels of three independent western blots for Fig. 6D.** LX-2 cells indirectly co-cultured with α-MEM, C-hADSCs-CM or P-hADSCs-CM, and the cells were collected for western blot. Lane 1: control group (control medium without TGF-β); Lane 2: α-MEM group (control medium added with TGF-β, 2ng/ml); Lane 3: C-hADSCs-CM group (conditioned medium from C-hADSCs added with TGF-β, 2ng/ml); Lane 4: P-hADSCs-CM group (conditioned medium from P-hADSCs added with TGF-β, 2ng/ml).

**
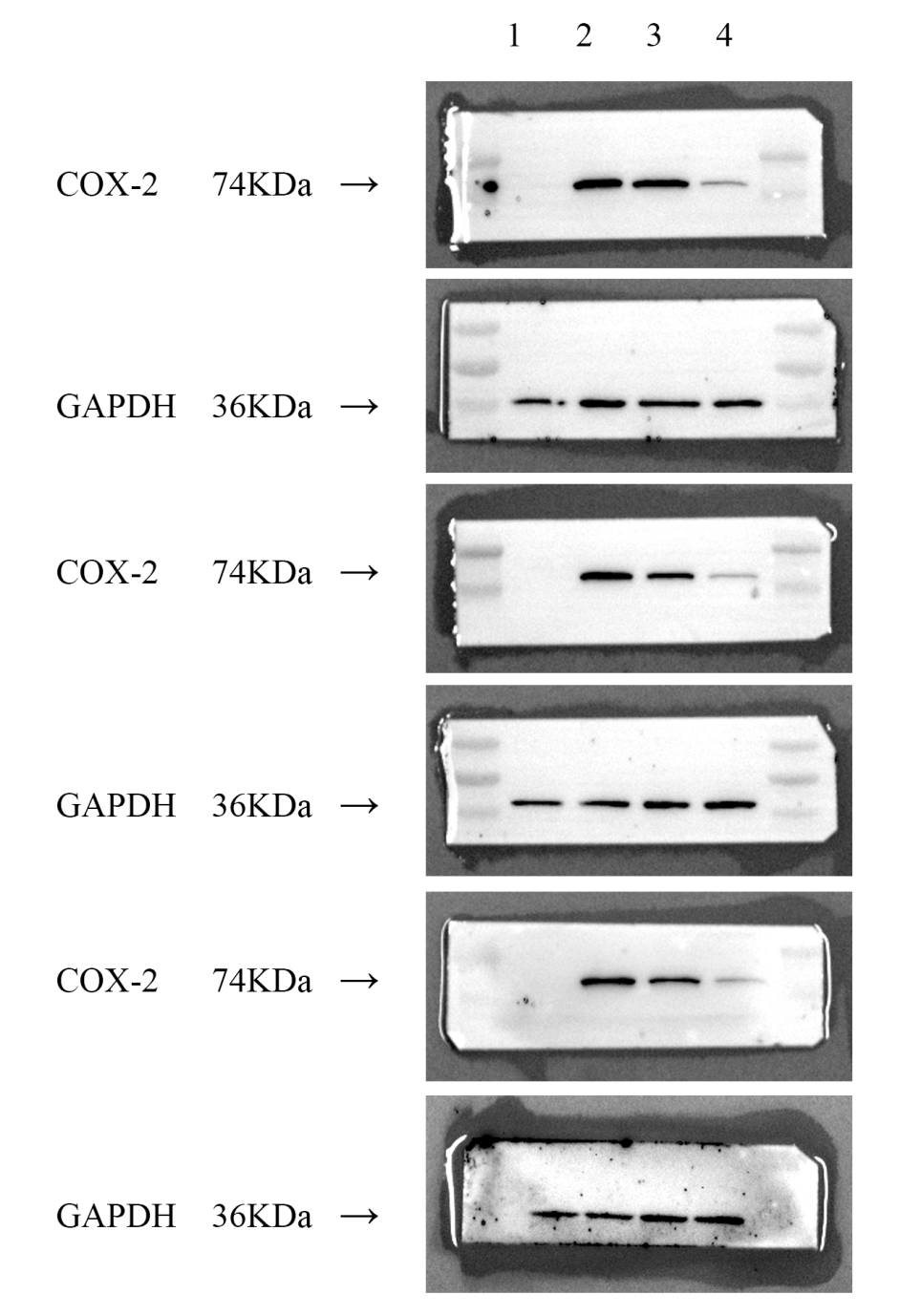
**

**Supplementary Figure 3. Full length gels of three independent western blots for Fig. 7B.** hADSCs pretreated with TNF-α and IL-1β for 0, 24, 48, and 72 hours. The cells were collected for western blot. Lane 1: 0h; Lane 2: 24h; Lane 3: 48h; Lane 4: 72h.

**
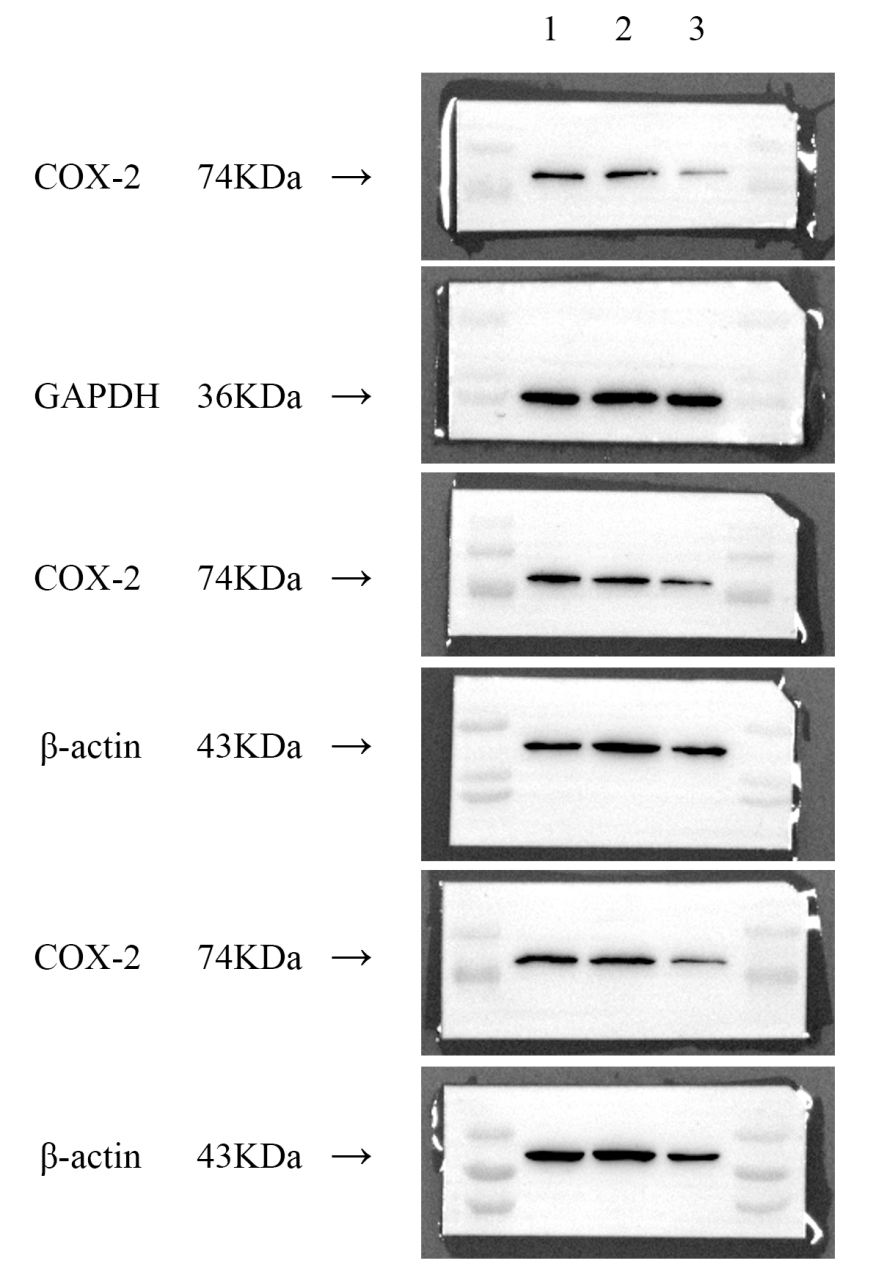
**

**Supplementary Figure 4. Full length gels of three independent western blots for Fig. 8A.** hADSCs transfected by COX-2 siRNA and the cells were collected for western blot. Lane 1: control group (untreated hADSCs); Lane 2: si-NC group (hADSCs transfected by control siRNA); Lane 3: si-COX-2 group (hADSCs transfected by COX-2 siRNA).

**
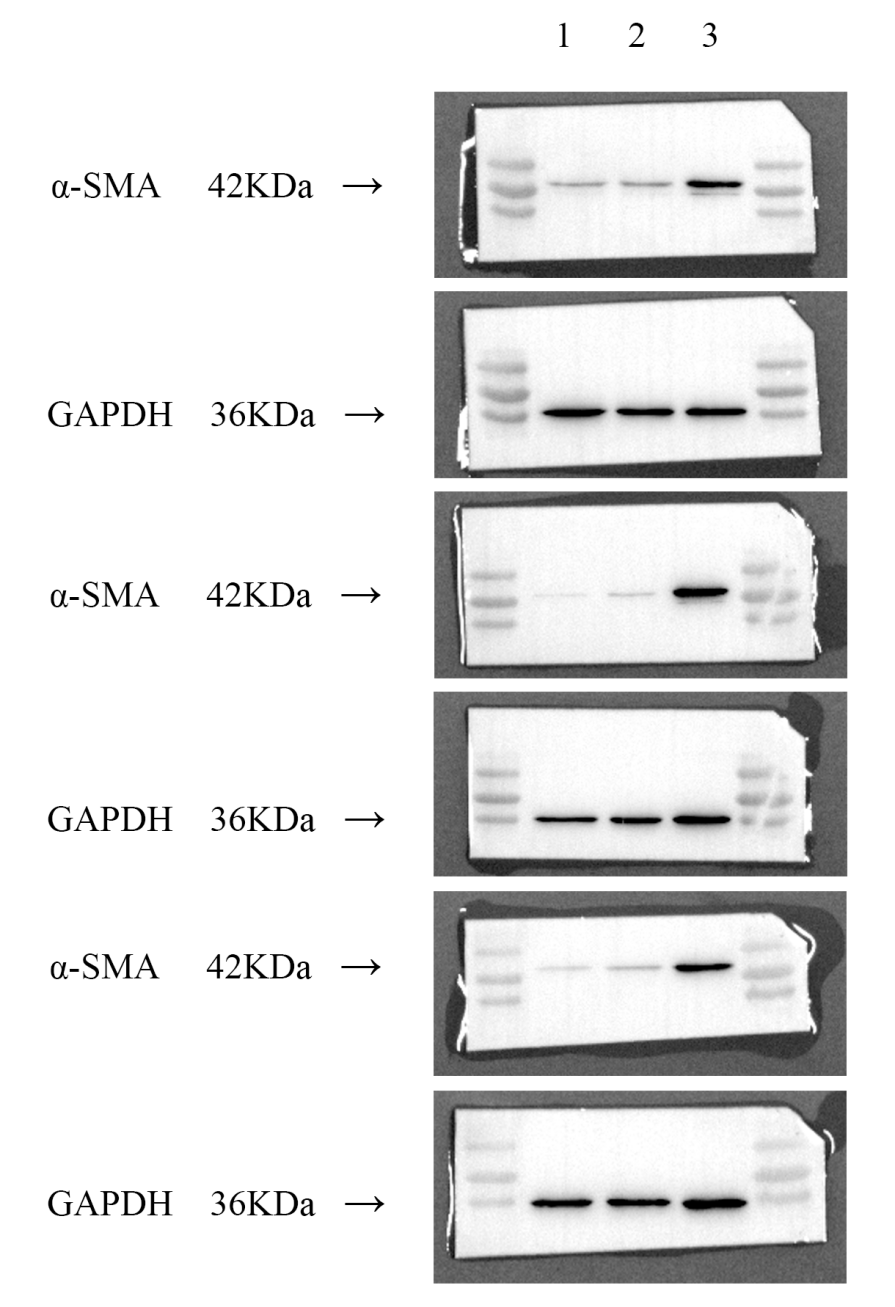
**

**Supplementary Figure 5. Full length gels of three independent western blots for Fig. 8E.** LX-2 cells co-cultured with conditioned medium from P-hADSCs, si-NC or si-COX-2, and the cells were collected for western blot. Lane 1: control group; Lane 2: si-NC group; Lane 3: si-COX-2 group.
